# Cross-Domain Transfer Learning for PCG Diagnosis Algorithm

**DOI:** 10.3390/bios11040127

**Published:** 2021-04-20

**Authors:** Kuo-Kun Tseng, Chao Wang, Yu-Feng Huang, Guan-Rong Chen, Kai-Leung Yung, Wai-Hung Ip

**Affiliations:** 1 School of Computer Science and Technology, Harbin Institute of Technology (Shenzhen), Shenzhen 518055, China; kktseng@hit.edu.cn (K.-K.T.); wangchao257@huawei.com (C.W.); 190110527@stu.hit.edu.cn (G.-R.C.); 2 School of Journalism and Communication, Xiamen University, Xiamen 361005, China; 3 Department of Industrial and Systems Engineering, The Hong Kong Polytechnic University, Hong Kong, China; kl.yung@polyu.edu.hk (K.-L.Y.); wh.ip@polyu.edu.hk (W.-H.I.)

**Keywords:** transfer learning, phonocardiogram, biosignal diagnosis

## Abstract

Cardiechema is a way to reflect cardiovascular disease where the doctor uses a stethoscope to help determine the heart condition with a sound map. In this paper, phonocardiogram (PCG) is used as a diagnostic signal, and a deep learning diagnostic framework is proposed. By improving the architecture and modules, a new transfer learning and boosting architecture is mainly employed. In addition, a segmentation method is designed to improve on the existing signal segmentation methods, such as R wave to R wave interval segmentation and fixed segmentation. For the evaluation, the final diagnostic architecture achieved a sustainable performance with a public PCG database.

## 1. Introduction

The development of machine learning has enabled great progress in the diagnosis of diseases using biosignals, and the diagnostic performance is constantly improving. The phonocardiogram (PCG) signal is a time-varying heart sound signal with periodicity. The PCG and ECG are the two most important biosensor signals for cardiovascular disease.

The main information in the PCG is within the frequency range of 0–800 Hz, the frequency range of human hearing is mainly between 20–20,000 Hz, and the most sensitive auscultation frequency is 1000–5000 Hz, so there is much important information in the PCG, but it is more difficult to classify compared to ECG.

The important pathological information contained in the PCG signal is not well used. Therefore, the design of an effective algorithm for the PCG signal that can better reveal the information than previous works is an important issue for automatic diagnosis of PCG.

However, mainstream algorithms have begun to move from conventional machine learning algorithms to deep learning algorithms. Compared with traditional machine learning, the main difference of deep learning lies in multiple hidden layers, and feature extraction from each layer is not involved in the design, but is self-learned from the data using a common learning process. In this way, scholars can focus more on improving the adaptability and classification accuracy of the model without spending a lot of effort on feature mining.

The conventional PCG is largely dependent on the doctor’s experience, and its reliability is not high. The main object of this work is to propose a deep learning framework for automatic diagnosis with a phonocardiogram. In summary, the contributions of this work are as follows:

(1) A new segmentation method is proposed to improve existing signal segmentation methods such as RR (R wave to R wave) interval segmentation and fixed segmentation.
(2) A large kernel is proposed to improve the convolution performance for the PCG waveform.
(3) Migration transfer learning is designed for this PCG framework, and can enable PCG migration from ECG to PCG.
(4) This paper proposes a boosting model based on Transfer Learning LKnet (MFS-LKNet Boosting).

The composition of this paper follows: Section 2 is related work, Section 3 is the architecture and algorithm, Section 4 is our expert results, Section 5 discusses some issues of this work, and the final section is our conclusion and references.

## 2. Related Work

The automatic diagnosis of heart sound is similar to that of the ECG. It is also divided into three parts: preprocessing, feature extraction, and classification recognition. For PCG diagnosis, many scholars have researched phonocardiograms, from noise interference removal, feature point recognition, feature parameter detection, waveform cluster analysis, waveform category judgment, and final diagnostic methods. The following describes the development status of PCG from four aspects: denoising, segmentation, feature extraction, and classification.

### 2.1. Noise Reduction

The PCG signal collected is generally composed of the heart sound and noise. The noise is generally divided into ambient noise and the noise of the instrument itself. Ambient noise is the noise in the environment in which the heart sounds are collected, and also contains respiratory sound. The noise generated when the instrument rubs against the skin is also an environmental noise. The instrument itself can also generate noise when collecting heart sounds due to the characteristics of its internal electronics. Ambient noise can be avoided artificially, instrument noise can also be reduced by improved devices, but it is still impossible to remove 100%, so a certain denoising step is required to make the useful components more prominent. PCG denoising essentially involves looking for a suitable filter to separate the wanted signal from the noise to provide a denoising effect. At present, the commonly used heart sound denoising methods include the empirical mode decomposition (EMD) denoising method, Hilbert yellow transform denoising method, and the wavelet denoising method [1,2,3].

### 2.2. Segmentation

The beat cycle segmentation is a very important part of the PCG automatic diagnosis research based on the feature extraction machine learning model. There are four main types of methods for heart sound segmentation [4]. (1) Envelope-based method—usually uses various techniques to construct an envelope of a heart sound signal, thereby performing heart sound segmentation. Typical methods for constructing envelopes include Shannon energy [5,6], Hilbert transform [7], extraction of heart sound signature waveforms [8], and square energy [9]. (2) Feature-based approach—this method requires the calculation of heart sound features and then segmentation decisions. Typical features include amplitude–frequency features [10,11], phase features [12], periodic component features [13], complexity-based features [14], multilevel wavelet coefficient features [15], and higher-order statistics such as kurtosis [16]. (3) The machine learning-based approach uses neural networks or other nonlinear classifiers that utilize extracted features, for example, a multilayer perceptron neural network [17], K-means clustering [18], dynamic clustering [19], and unsupervised learning methods [20]. (4) Hidden Markov Model (HMM) were originally proposed by Gamero and Watrous [21] in the context of heart sounds. Ricke et al. [22] subsequently developed this method. Then, Gill et al. incorporated timing duration and homomorphic filtering into the HMM method to improve the segmentation accuracy [23,24], and Schmidt et al. extended HMM to a hidden semi-Markov model (HSMM) by modeling the expected duration of heart sounds [25]. Springer et al. [26] further developed the HSMM method by using additional input features, logistic regression, and an improved Viterbi algorithm, which solved the boundary conditions and improved the overall segmentation of short-term heart sound recording.

### 2.3. Feature Extraction

The features that the PCG can extract include time-domain features such as mean, variance, area, and amplitude, and various frequency domain features, such as the early-time short-time Fourier transform (STFT) [27,28]. The works use STFT to construct a time-varying transfer function and a time-varying coherence function that relate the heart and chest heart sounds; Ref. [28] used STFT to identify congenital heart disease such as atrial septal defects, ventricular septal defects, and arteriovenous catheterization. STFT reduces the signal stability requirements, is easy to implement, and is easy to calculate. However, its time–frequency window is fixed in the entire time–frequency plane, which cannot meet the requirements of different window functions for different components. Wavelet transform can realize the time-varying spectrum analysis of signals [29], and can analyze the details of the signal in more detail, which is the most widely used heart sound analysis method. In addition, empirical mode decomposition, Mel frequency cepstral coefficients (MFCC), linear predictive cepstral coefficients, etc., can also be used for PCG feature extraction [30,31,32].

### 2.4. Diagnostic Classification

Models for ECG classification can be used for PCG, such as SVM, and BP neural network. Jiang et al. [33] used normalized autoregressive power spectral density (NAR-PSD) curves and support vector machine (SVM) techniques to classify cardiac noise. They obtained 459 heart sound signals from six healthy volunteers and 30 patients, including 196 normal and 263 abnormal sound cases, and finally achieved high classification performance. Leung [34] used a neural network classifier to classify heart sounds; and Turkoglu [35] designed an expert system based on a BP neural network to successfully diagnose heart valve disease.

Deep learning studies for PCG diagnosis have brought more possibilities and higher accuracy to PCG classification diagnosis. Potes et al. [36] used the feature-based AdaBoost model classifier and Convolutional Neural Network (CNN) model classifier to classify on the open PCG database, and obtained an average accuracy of 0.79 and 0.82, respectively, with a rate that reached 0.86. Sujadevi et al. [37] demonstrated the feasibility of RNN and its variants LSTM and GRU in the diagnosis of PCG classification.

## 3. Architecture and Algorithm

**Figure 1:**
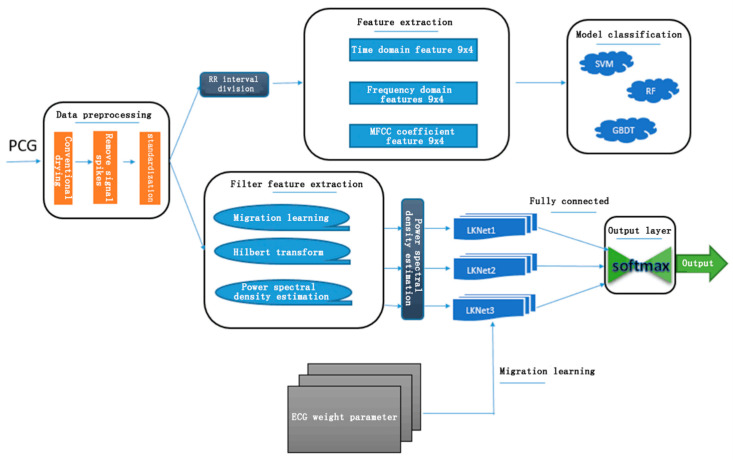
Framework for phonocardiogram (PCG) algorithms.

The entire diagnostic system includes the following modules: noise reduction and spike removal, signal segmentation, feature extraction, and model classification. Noise has power frequency interference, myoelectric interference, and baseline drift. The signal segmentation method is based on the ECG segmentation method, and is further divided into several short waves, which are the four short-wave states of S1, Systole, S2, and Diastole. Feature extraction based on a traditional machine learning model classification includes 36 time domain features such as mean and variance of the PCG interval and amplitude, 36 frequency domain features based on Hamming window and discrete time Fourier transform, and 52 MFCC coefficient features. For these features, the three classification models of Support Vector Machines (SVM), Random Forest (RF) and Gradient Boosting Decision Tree (GBDT) are used here. The features based on the deep learning neural network are extracted by homomorphic filtering, Hilbert transform, and power spectral density estimation, and the classification model introduces the concept of migration learning. The entire classification system structure is shown in Figure 1.

### 3.1. PCG Noise Reduction and Removal of Spikes

There is also some noise in the PCG signal, which is inevitable during the acquisition process. Like the ECG signal, the PCG noise can be removed to some extent by the Butterworth filter. Since the frequency of the PCG signal in the data set is 0–2000 Hz, most single-sample PCG signals are also longer in length, so downsampling is first performed to reduce the frequency to below 1000 Hz. The Butterworth band-stop filter is then used to remove the low frequencies in the signal, i.e., to remove signals below 25 Hz and signals above 400 Hz, which ensures that important information is substantially preserved while effectively removing most of the noise.

The PCG signal may have some signal spikes that interfere with the model classification. The algorithm for removing signal spikes mentioned by Schmidt in [25] is mainly for PCG signals. The peak signal in the data set used in this experiment appears less, but since the time complexity of this algorithm for removing spikes is small, the actual experiment does not take up significant time, and it is also reserved.

### 3.2. PCG Signal Segmentation

**Figure 2:**
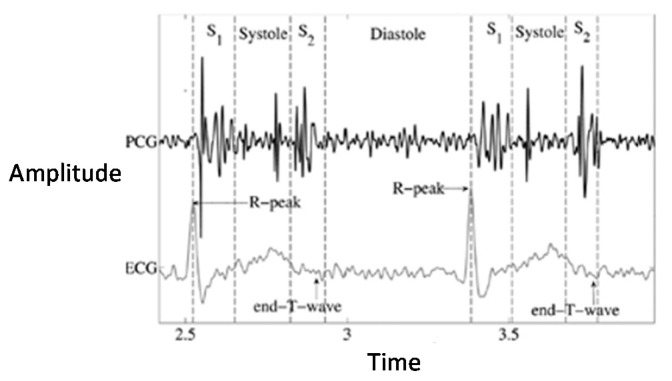
Waveform of PCG.

There are many mature PCG signal segmentation methods. Chen et al. [38] mainly use K-means cluster analysis for PCG segmentation [39] and use neural networks to segment Fundamental Heart Sounds (FHS) between S1 and S2. Because of the PCG dataset used in this chapter, some samples contain ECG data collected at the same time, and the segmentation effect of ECG signals is very good and the accuracy is extremely high. Therefore, the semihierarchical Markov chain segmentation method proposed in [26] is adopted. After the ECG signal is segmented, the corresponding point is migrated to the corresponding PCG signal as a training set. Figure 2 is a diagram of the heart sound signal and the ECG signal corresponding to the same heart beat information.

The specific steps are as follows:

(1) The signal is divided into 500 ms per window.
(2) Find the maximum absolute amplitude (MAA) in each window.
(3) If at least one MAA exceeds three times the median of all window MAAs, perform the following steps. If not, go to step (4).
  - Select the window with the largest MAA value.
  - In the selected window, the position of the MAA point is defined as the top of the noise spike.
  - The beginning of the noise spike is defined as the last zero crossing point before the MAA point.
  - The end of the peak is defined as the first zero crossing point after the maximum point.
  - The defined noise spike is set to zero.
  - Go back to step (2) and continue.
(4) The program is complete.

In the data set used in the experiment in this paper, the occurrence of spike signals is less frequent, and because the time complexity of the spike removal algorithm is still small, it is also reserved.

### 3.3. Filter Feature Extraction

The deep learning algorithm itself has its own feature extraction capability, which can directly feed the continuous state samples that are related before and after. After the ECG signal is preprocessed, some continuous transformations can be performed to convert the signal into other states that can be interpreted, such as spectral transform. Through these feature transformations, it is possible to capture the feature information implied by the original information into other spaces or at other levels, while maintaining the continuity of the features themselves.

Through the deformation of the heart sound signal, this paper mainly extracts three types of continuous feature models for deep learning. The methods adopted are homomorphic filtering, Hilbert transform, and power spectral density (PSD) estimation.

a. Homomorphic filtering is often used for image processing and is a kind of frequency domain filtering. However, homomorphic filtering has its own advantages. Frequency domain filtering can flexibly solve the additive noise problem, but it cannot reduce multiplicative or convolution noise. In order to separate the additive combination signal, the linear filtering method is often used. The nonadditive signal combination commonly uses the homomorphic filtering technique to convert the nonlinear problem into a linear problem processing, that is, first to nonlinear (multiplicative or convolution). The mixed signal is a mathematical operation that is transformed into additive. Then, it is processed by the linear filtering method, and finally the inverse transform operation is performed to restore the processed image.

In image processing, it is often found that the dynamic range is large but the details of the dark area are unclear. We hope to enhance the dark area details without losing the details of the bright area. In general, we can image *f*(*x*, *y*) modeled as the product of the illumination intensity *i*(*x*, *y*) and the reflection intensity *r*(*x*, *y*):

(1) f(x,y)=i(x,y)r(x,y)

In general, the illumination of natural pictures is uniform, so *i* should be a low-frequency component, and the reflection of light by different objects has abrupt changes, so *r* is a high-frequency component. Now take the logarithm of the two sides and perform the Fourier transform to get the frequency domain of the linear combination:

(2) lnf(x,y)=ln(i(x,y))+ln(r(x,y))

(3) FFT(lnf(x,y))=FFT(ln(i(x,y)))+FFT(ln(r(x,y)))

After the operation is completed, the inverse transformation is performed, and the processed image is obtained by exponentiation. Using this for heart sounds can reduce the low-frequency detail and pull up the high frequency.

b. Hilbert transform is a common method in signal processing. It has a real-valued function *x*(*t*) whose Hilbert transform is denoted as $\hat{x}\left(t\right)$ or *H*[*x*(*t*)] as follows:
  (4) $$x\left(t\right)=H\left[x\left(t\right)\right]=-\frac{1}{\pi }\overset{\infty }{\underset{-\infty }{\int }}\frac{x\left(\tau \right)}{t-\tau }d\tau$$

In contrast to the concept of convolution, it can be found that the expression of the Hilbert transform given above is actually the result of convolving the original signal with a signal. Therefore, the Hilbert transform can be thought of as passing the original signal through a filter, or a system whose impulse response is *H*(*t*). Performing a Fourier transform on *H*(*jω*), one can get the following:

(5) H(jω)=−jsgn(ω)

c. Power spectral density estimation:
  (6) $$P_{X}\left(e^{j\omega }\right)=\overset{+\infty }{\underset{m=-\infty }{\sum }}r_{X}\left(m\right)e^{-j\omega m}$$
  where *r_x_* (*m*) is the autocorrelation function of the random signal. The power spectrum reflects that the power of the signal is distributed in the frequency domain with frequency ω, so *P_x_* (*e^jω^*) is also called the power density:
  (7) $${\hat{P}}_{AC}\left(\omega \right)=\overset{M}{\underset{m=-M}{\sum }}\hat{r}\left(m\right)e^{-j\omega m},\;\;\;\left|M\right|\leq N-1$$

Because the power spectrum obtained by this method is obtained indirectly through the autocorrelation function, it is called an indirect method, also known as an Auto-Correlation (AC) method. When *M* is small, this formula is not very large, so this method was a commonly used spectral estimation method before the *FFT* was introduced (that is, before the periodogram method was widely used).

### 3.4. Transfer Learning LKNet

**Figure 3:**
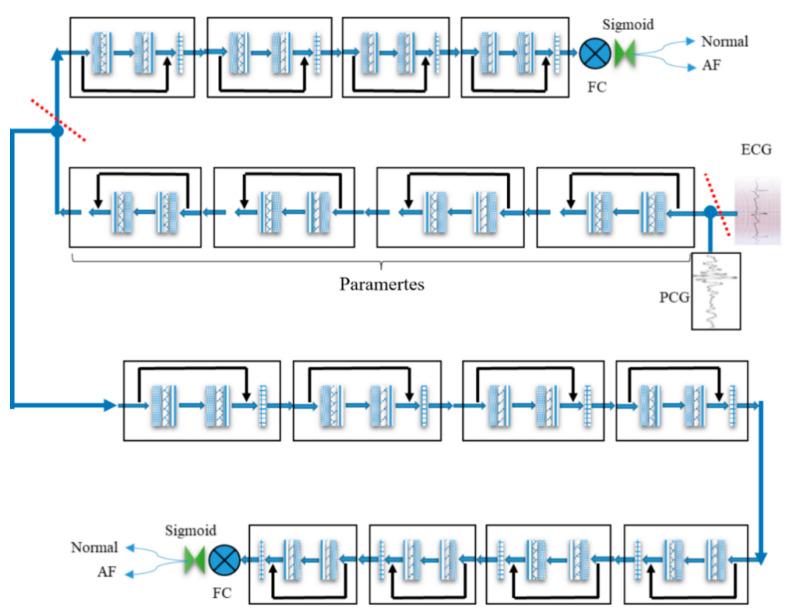
The transfer learning of large kernel network LKNet processes from ECG to PCG.

This paper proposes a transfer learning architecture with a large kernel network (LKNet) as shown in Figure 3. LKNet is a large-size convolution network based on a residual network, which is called transfer learning LKNet. Because ECG data are more common and has cardiac activity related data with PCG, this algorithm uses a transfer learning method to migrate knowledge from the ECG to the PCG network.

Transfer learning is to move the knowledge of one domain (i.e., the source domain) to another domain (i.e., the target domain), so that the target domain can achieve better learning results. Usually, the amount of data in the source domain is sufficient, and the amount of data in the target domain is less. This scenario is very suitable for migration learning. For example, we have to classify a task, but the data in this task are not sufficient (target domain), although there is a large amount of relevant training data (source domain); these training data are different from the test data of feature distribution in the classification task that needs to be performed. In this case, if the appropriate transfer learning method can be adopted, the sample is not sufficiently improved.

Transfer learning is widely used in the field of image classification. Usually, a super-large neural network that has been trained is regarded as a source domain, and a model network specific to small sample image data is used as a target domain. At this time, transfer learning is more effective; for example, ResNet101 trained in the ImageNet dataset is used to classify the small sample cat and dog image data collected by itself. Generally speaking, the more source domain samples there are, the fewer target domains there are relative to the source domain. The more similar the samples are, the more obvious the improvement in accuracy is compared to the migration of the target field samples.

The PCG is stored by conversion into audio, and the ECG is stored by conversion into an electrical signal. These two signals, however, are strictly different signals in different fields, which mean that the similarity is not high enough to be reminiscent of migration learning. Nevertheless, under the existing conditions, through the analysis of the database, the ECG data are relatively clean, the classification effect is very good, and the ECG data sample size is several times that of the PCG, and then the combination of their collection source and the target are the characteristics of the heartbeat. In order to explore the commonality between ECG and PCG signals in deep learning, and the generalization of the improved model LKNet, the migration learning experiment was conducted.

As shown in Figure 3, the specific process follows:

(1) From the Normal, Atrial Fibrillation (AF) Abnormal, Noise, and Other types of data samples in the ECG database, Noise and Others are removed, and the retained Normal and AF are exactly the same as the sample categories in the PCG database.
(2) The ECG data are first input to Full Connected (FC), than it is trained by LKNet, and eight blocks are used to obtain a source network with an accuracy of 90% or more.
(3) The last four of the eight blocks in the source network are removed, then the first four are fixed, and eight blocks are added to form a new network for training PCG data.
(4) Figure 3 shows the process for the PCG migration from ECG data.

### 3.5. Boosting LKNet

In order to further improve the accuracy, we can fuse multiple LKNets to boost the transfer learning algorithm described above. The base classifier of the boosting algorithm model is generally a weak classifier. Since a single weak classifier is generally underfitting to the training sample, the model lifting effect is better. However, deep learning models are generally strong classifiers and have the ability to overfit the training set. The boosting model is very sensitive to the abnormal sample, because the input of each classifier in the boosting model depends on the classification result of the previous classifier, which results in exponential error accumulation. The sample size used for deep learning model training is large and a certain degree of mislabeling is allowed, which will seriously affect the performance of the boosting model. Therefore, boosting is usually not combined with deep learning models.

**Figure 4:**
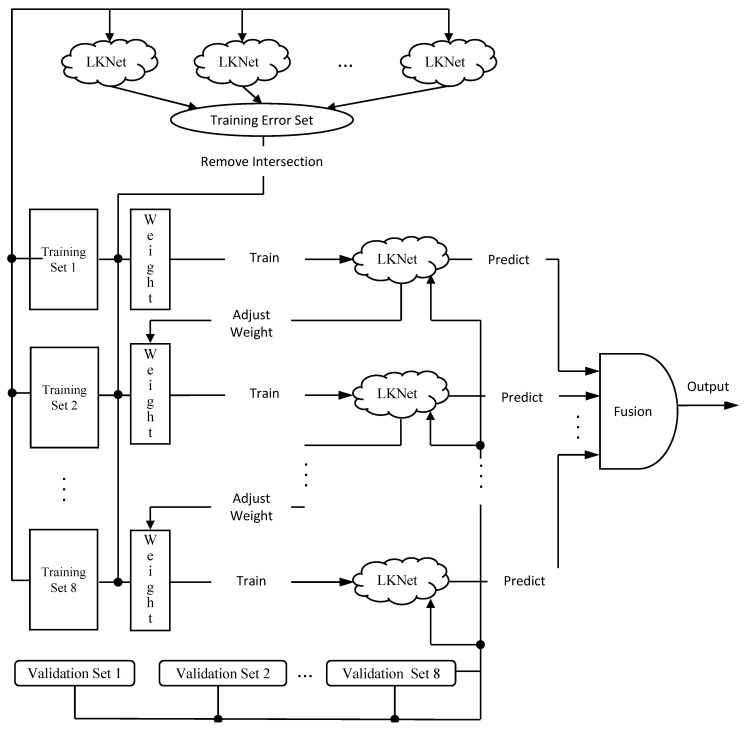
The multifeature sets (MFS)-LKNet Boosting.

However, Figure 4 shows the main idea of the proposed model. There is some research on PCG in this section that can be applied to boosting methods. This section proposes a multifeature set and CNN-based boosting model (multifeature sets LKNet Boosting, MFS-LKNet Boosting). There are two reasons for the proposed boosting algorithm.

(1) The first reason is that PCG has fewer public databases. The database selected this time is relatively large, but there are fewer than 3300 samples, and PCG can extract several continuous features, such as state filtering, Hilbert transform, PSD features, and others. It is helpful for the boosting model to learn the characteristics of PCG signals from different angles.
(2) The second reason is that LKNet is adopted from CNN, the structure proposed in the previous section, and it has ideal performance for single modules. Therefore, the method of our boosting classifier enhances the predictive ability through multiple loop iterations of weight adjustment. The iterative optimization task is mainly completed by adjusting the weights of LKNet, and only a small portion is carried out by the fusion module.

## 4. Experiment

### 4.1. Data Sources and Evaluation Criteria

The PCG data used in this study are from the world’s largest physiological signal library website (www.physionet.org, accessed on 15 April 2021), the public database used for Computing in Cardiology (CinC) challenge 2016. There are two kinds of classification tags, abnormal and normal, and the evaluation standard is the mean of specificity and sensitivity (MAcc).

### 4.2. Comparison of Different Segmentation

Based on the similarity of data structures between PCG and ECG, and the similarity of the purpose of disease diagnosis based on them, the deep learning model classification part of this work directly uses LKNet. The operation of the sliding window segmentation method previously proposed for the ECG signal does not bring about a significant change in accuracy. The Table 1 shows the results of the experimental classification.

When the stride and the window are equal, the sliding segmentation becomes a fixed segmentation. The table below shows the results of the window segmentation, fixed segmentation, RR interval segmentation and nonsegmentation. The final results of the three methods of segmentation and the nonsegmentation are almost the same, and the effect of sliding window segmentation is obviously better. Since the length of most PCG signal samples does not exceed 1 min, the window is set to 10 s. The best Mean Accuracy of Percentage (MAcc%) is 79.25 for sliding segmentation with stride 1 and window(s) 2.

### 4.3. Comparison of Single Classification Method

In this subsection, the classification methods with single features compared in this experimental method are Random Forest (RF), Support Vector Machine (SVM), and Gradient Boosting Decision Tree (GBDT), which are the current mainstream machine learning methods. At present, the mainstream deep learning algorithm is the Convolution Neural Network (CNN). Our proposed Large Kernel Network (LKNet) has several versions of improvement, such as LKNet with Homo, Hilbert, Power Spectral Density (PSD), LKNet with ensemble SVM, and LKNet with transfer learning. Among them, transfer learning gives the best performance. The detailed experimental results are shown in Table 2.

It can be seen from Table 2 that, after the PCG signal undergoes homomorphic filtering, Hilbert transform, and PSD estimation, the extracted feature is added to LKNet and the MAcc value is 80.16, which is 1% higher than the original signal input. The addition of transfer learning has increased the model classification results by more than two percentage points. Both PCG signal transformation and transfer learning can bring about an improvement in the model classification accuracy. The traditional machine learning method linear SVM used in this paper has relatively low precision, although many features have been extracted through feature engineering. If we only consider the single module, our transfer learning LKNet has achieved relatively good results.

### 4.4. Comparison of Fusion Classification

Observing Table 3, Homsi et al. [40] used traditional classifiers based on feature extraction to perform model fusion through Random Forest (RF), LogitBoost (LB) and Cost Sensitive Classifier (CSC), and finally reached a MAcc value of 88.4. The second model compared comes from the fusion model of Potes et al. [36], whose fusion method uses a threshold to select from CNN or AdaBoost classification results and reach the previous high MAcc value of 89%. The last one compared, Tang et al. [41], extracted as many as 515 features, including temporal features in nine categories, namely sign, amplitude feature, energy feature, cepstrum feature, cyclostationary feature, high-order statistical feature, and cross-entropy feature, and then used SVM for fused classification, and reached a MAcc value of 88%. The boosting algorithm based on MFS LKNet proposed in this paper benefits from the good performance of transfer learning LKNet in the previous subsection, and the MAcc value increases by 1% on this basis. Compared with the latest paper with the highest accuracy, there is a 2% improvement, achieving the effect of state-of-the-art.

## 5. Discussion

In this section we discuss two macro issues and two micro issues of this research. The macro issues are algorithm and application contribution, the micro issues are data set and evaluation procedure of this work.

1. What Is the Algorithm Contribution of this Work?

Compared with the transfer learning of images and texts, there is less research on the cross-domain transfer learning of biological signals. In a summary, our algorithm contribution includes proposing sliding window segmentation, LKNet with transfer learning, and LKNet with feature fusion algorithms. For major contribution of cross-domain transfer learning, this research preliminarily proved that two different but similar biological signals can also be transferred learning to improve classification accuracy. In addition, we conducted an in-depth study on the PCG algorithms, which can support PCG researchers to conduct further research with deep learning algorithm.

2. What Is the Application Contribution?

In terms of an application contribution, because heart sound is a diagnostic device commonly used by physicians, this research provides broad practical application value and can assist physicians in making more accurate diagnosis. With the mature development of smart phone, however, our algorithms can be used on mobile devices. In this way, ECG and PCG can be integrated into a separate mobile device, or integrated into a mobile phone. The inference model can be placed on the mobile phone for automated diagnosis, allowing users to collect diagnostic data from time to time, which can make up for the shortcomings of hospital service that are not in a timely manner.

The major value of this research is to explore the possibility of transfer learning between similar heterogeneous multimodal biosignal data. According to our research results, this type of heterogeneous data should be able to learn from each other’s experience. We hope that our results can provide some references for the future research.

3. What Is the Data Set?

Our data set is a public ECG/PCG data set of CINC challenge. This PCG data set includes five groups of 3126 heart sound records from 764 subjects, with a sound duration ranging from 5 to 120 s. ECG recordings, collected using the AliveCor device contains 8528 single lead ECG recordings lasting from 9 to 60 s.

As the scope of the selection of input data for our given research problem, our design goal is to realize our algorithm with mobile ECG and PCG equipment. Therefore, we choose the data set by CINC’s mobile devices. However, as CINC’s ECG and PCG are not collected at the same time, it may be such an impact on the performance that the improvement of transfer learning is not enough. In the future, if data are collected at the same time, the effect of transfer learning should be better.

4. What Is the Evaluation Procedure?

The test procedure is focused on the accurate classification of normal and abnormal PCG. The training and testing sets are each divided into two sets of mutually exclusive populations. The testing set is approximately 10% of the training set, which is a copy of 300 records from the training set. The evaluation is carried on a 10-fold cross-validation with training and testing sets. Based on this, we summarize the overall evaluation process as follows:

Step 1: Divide the data into six training and test sets;
Step 2: First train separately for ECG data set and PCG data set;
Step 3: Transfer the training weight of ECG to the PCG network;
Step 4: Select the test group that is the different as the training set and perform the test;
Step 5: Repeat Step 2 for the cross-validation experiment.

## 6. Conclusions

In this paper, we successfully propose a PCG diagnosis classification algorithm, and make some improvements, including sliding window segmentation, LKNet with transfer learning, and LKNet with feature fusion algorithms. The experiment is based on a PCG automated diagnostic framework followed by frame steps for signal denoising and spike removal, and sliding window segmentation. For the deep learning feature extraction part, the feature extraction method in the image domain is also used for reference. The MFCC coefficient is also added, based on the machine learning feature extraction. The previously proposed sliding window segmentation method is better applied in this paper. At the same time, it also introduces the concept of transfer learning, designing a set of processes, and successfully applying the characteristic parameters learned from ECG to the PCG diagnostic classification. Finally, this paper proposes a multifeature set with the LKNet-based boosting model, which achieved the best result compared with state-of-the-art methods.

In the future, this research will advance in two directions. On the one hand, the algorithm will continue to be enhanced to improve its accuracy. On the other hand, the algorithm can be applied to other time-series biological signals to enable this research to make a greater contribution.

## Figures and Tables

**Table 1:** Comparison of segmentation methods.

Segmentation Method	Stride (s)	Window (s)	MAcc (%)
Fixed Segmentation	2	2	77.38
Sliding Segmentation	1	2	79.25
	2	5	78.14
	4	10	79.06
Beat Segmentation	-	-	77.21
No Segmentation	-	-	77.64

**Table 2:** Comparison of single classification methods.

Models	MAcc (%)
Linear SVM	66.48
RF	66.42
GBDT	65.01
LKNet	79.06
LKNet with Homo, Hilbert, PSD	80.16
LKNet with Ensemble SVM	71.39
LKNet with transfer learning	82.73

**Table 3:** Comparison of fusion methods.

Paper	Model	Sensitivity (%)	Specificity (%)	MAcc (%)
Homsi et al. [40]	Fused RF + LB + CSC	94.4	86.9	88.4
Potes et al. [36]	Fused AdaBoost + CNN	96	80	89
Tang et al. [41]	Fused 515 Features SVM	88	87	88
Our paper	MFS-LKNet Boosting	96.34	86.62	92.48

## Data Availability

The raw/processed data required to reproduce these findings cannot be shared at this time as the data also forms part of an ongoing study.
